# Sweeteners in E-Cigarettes: A Minireview of Flavoring and Biological Action

**DOI:** 10.3390/jox15060209

**Published:** 2025-12-11

**Authors:** Volodymyr V. Tkach, Tetiana V. Morozova, Isabel Gaivão, Ana Martins-Bessa, Yana G. Ivanushko, José Inácio Ferrão de Paiva Martins, Ana Novo Barros

**Affiliations:** 1 Engineering Faculty, University of Porto, Rua Dr. Roberto Frias, s/n, 4200-065 Porto, Portugal; jipm@fe.up.pt; 2 Centre for the Research and Technology of Agro-Environmental and Biological Sciences (CITAB), University of Trás-os-Montes and Alto Douro (UTAD), 5000-801 Vila Real, Portugal; 3 Animal and Veterinary Research Center (CECAV), Associate Laboratory for Animal and Veterinary Sciences (AL4AnimalS), University of Trás-os-Montes and Alto Douro (UTAD), 5000-801 Vila Real, Portugal; igaivao@utad.pt (I.G.); abessa@utad.pt (A.M.-B.); 4 State Scientific Institution “Institute of Ecological Restoration and Development of Ukraine”, Environment Remediation Section, Mytropolite Vasyl Lypkivsky Str. 35, 01001 Kyiv, Ukraine; tetiana.morozova@ukr.net; 5 Disaster and Military Medicine Department, Bukovinian State Medical University, Teatralna Sq. 9, 58001 Chernivtsi, Ukraine; yana_iv@ukr.net

**Keywords:** electronic cigarettes, natural sweeteners, artificial sweeteners, aspartame, saccharin, sucralose, oxidative stress, genotoxicity, environmental effects

## Abstract

The use of sweeteners in e-cigarette liquids has become increasingly common, aiming to enhance the sensory appeal of vaping products. Compounds like aspartame, saccharin, and sucralose are added to provide a sweet taste without any calories, especially in flavored e-liquids popular among younger users. However, recent studies suggest that these additives may pose significant health risks when vaporized and inhaled. Sucralose, in particular, can break down into potentially harmful chlorinated by-products at high temperatures typical of vaping devices. Moreover, there is growing concern about the synergistic effects of sweeteners like sucralose, one sweetener with another and when combined with other e-liquid components. It has been observed that the presence of sucralose may amplify oxidative stress; genotoxicity, including mutations; and overall toxicity, along with environmental impact. This is not limited to nicotine- and smoke-related harm, as it may strengthen the toxic effect of the substances used in e-liquids that are not present in traditional cigarettes. The combined exposure to these heated compounds can intensify cytotoxicity, potentially increasing the risk of respiratory, cardiovascular, and neurological effects over time. While marketed as safer alternatives to tobacco, e-cigarettes containing sweeteners like sucralose may introduce new and poorly understood toxicological hazards that deserve urgent regulatory attention.

## 1. Introduction

The global rise in electronic cigarette use has reached alarming proportions, becoming what many public health experts now call a silent pandemic [1]. E-cigarettes have quickly become highly popular among both older and younger people [2,3] including teenagers, and this popularity has been based on a contradictory statement that they are an allegedly safe alternative to common cigarettes. This statement, accepted without full awareness of the risks e-cigarette components may pose [4,5], is at least partially wrong.

Smoking among teenagers had already been a problem during both the XX and XXI centuries [6,7,8]. Nevertheless, this problem persists, as students in the early years of secondary school are now among the frequent users of vaping devices [9,10], which indicates the decreasing age of smoking initiation in the ages of Z and alpha, and this is probably the most troubling factor of vaping use. This trend raises serious concerns given the vulnerability of adolescent neurological development and behavior transformation, leading to a high potential for long-term addiction and harm [11,12].

**Figure 1:**
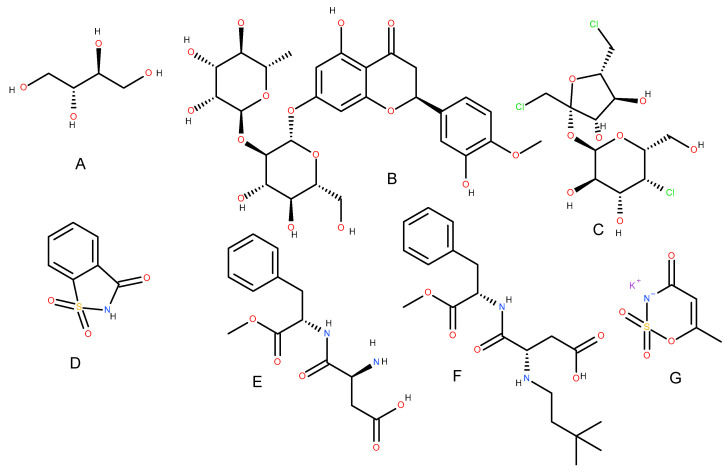
Erythritol (**A**), neohesperetine (**B**), sucralose (**C**), saccharin (**D**), aspartame (**E**), neotame (**F**), and acesulfame-K (**G**).

One of the main factors contributing to the popularity of e-cigarettes among youth is the wide variety of sweet, fruity, and candy-like smells and flavors, aimed to hide the bitter taste of nicotine and other smoking product flavors, while they face the conversation with parents and authority [13,14]. These are often made palatable and appealing through the addition of high-intensity sweeteners, including either natural (erythritol, perillartine, stevia, or neohesperidin) or synthetic (sucralose, saccharin, aspartame, neotame, advantame, and acesulfame potassium) sweeteners, creating the illusion of a somehow smoother and more attractive inhalation experience (Figure 1) [15,16]. Natural sweeteners based on polyphenolic and some terpenoid compounds may act as antioxidant and anti-inflammatory agents due to their chemical structure, providing, directly or indirectly, the chain termination of the radical process; other natural and synthetic sweeteners are much less biocompatible and toxic. Moreover, their interaction leads to a much more negative synergetic effect, which is still pending a thorough investigation [17,18,19,20].

The work [18], for example, mentions the toxic effect of different sweeteners, which may be manifested more expressively when the e-liquid is inhaled, as shown in [19,20].

While many of these sweeteners are approved for oral consumption at normal temperatures, their safety profile changes drastically when heated and inhaled [18,21,22] due to metabolic differences in digestive and respiratory systems. Unlike oral intake, inhalation can deliver these substances and/or their thermal degradation products, formed in the e-liquid or its aerosol, directly into the lungs, bypassing digestive metabolism and exposing delicate epithelial tissues to chemical degradation products [23,24]. Moreover, these compounds tend to provoke oxidative stress in epithelial cells, leading to direct [25,26] and indirect cytotoxic and genotoxic effects [27,28], which are still a lacuna to be fulfilled.

Among the most widely used sweeteners, sucralose, a chlorinated galactosucrose derivative, deserves special attention [29,30], being a chloroorganic compound, parent [31,32] to those already found in cigarette smoke [33,34]. Firstly, sucralose samples present in vapes may contain its industrial precursor, 6-acetylsucralose, which has proven to be genotoxic [35,36]. Moreover, when heated to the temperatures typical of vaping devices, sucralose can decompose into chlorinated compounds, including chloropropanols, dioxins, polychloroarenes, and 6-desoxychlorofrutctose, which may be even more harmful than the proper chloroorganic sweetener. Moreover, the enhanced use of these compounds in vapes may lead to environmental stress [37,38]. The review article [35] lists the effects of the environmental, oxidative, and genomic stress sucralose may pose. Aspartame, advantame, and neotame derivatives are also toxic at high temperatures, as they may yield methanol during their decomposition and hydrolysis [39,40,41,42]. The same concerns are also referent to saccharin. For this reason, the sweeteners’ biological behavior in e-cigarettes deserves special attention.

**Figure 2:**
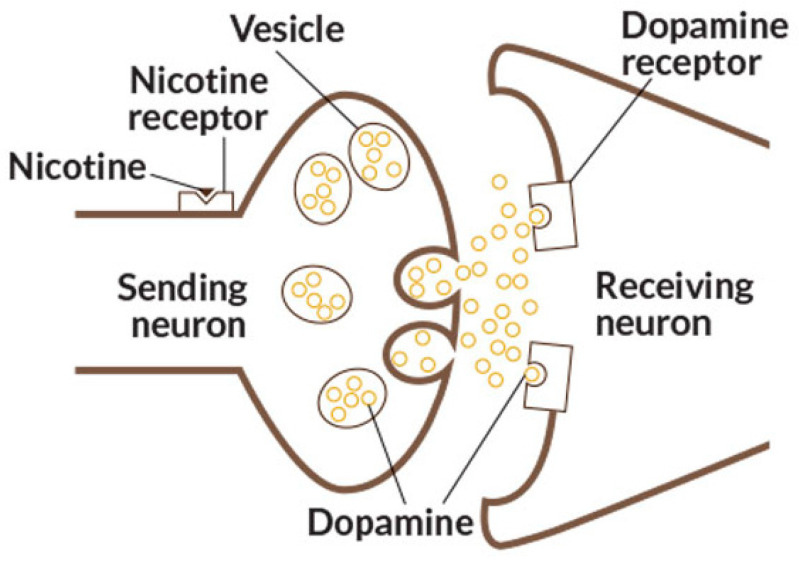
Nicotine interaction with the brain receptor. Image from Wikimedia Commons, licensed under CC BY 4.0.

Moreover, unlike conventional cigarettes, which contain a relatively fixed mixture of tobacco-related compounds (which inclusively makes them more fool-proof than vapes), e-liquids are chemically diverse and often include non-typical substances absent from combustible tobacco [43,44,45]. Also, the proper nicotine derivatives and other *N. tabacum* and *N. rustica* alkaloids may be present in them in more water-soluble and, as a result, more biocompatible salt form, acting directly on brain nicotine receptors (Figure 2), provoking dopamine secretion and brain stimulation, leading an addicted user to continue smoking for hours.

The heating and aerosolization of these complex mixtures may produce unknown or poorly studied by-products [46,47], posing significant toxicological risks when inhaled chronically.

The detailed information concerning the chemical composition of e-cigarette liquids may be consulted in Appendix A of the present article and the references therein.

Despite growing evidence, regulation of sweeteners in e-cigarette products remains lax or entirely absent in many jurisdictions [48,49]. Even EFSA and US FDA regulate the sweeteners’ presence mostly in food but not in electronic liquids and vaping devices, which indicates the overall or nearly overall lack of attention to the problem. Manufacturers are often not required to disclose the full list of ingredients, which makes it difficult to determine the role of e-liquid component interactions in the general health of smokers and the environment around them. For this and other reasons, the long-term health effects of inhaling sweetened aerosols are still not well understood [50].

In this context, the present study aims to critically examine the role of sweeteners—particularly sucralose—in the formulation of e-liquids, their biological action in human and other organisms, and also their environmental fate. It explores the mechanisms of thermal degradation, potential for synergistic toxicity caused by the interaction between two sweeteners or between the sweeteners and the main e-liquid components, and the broader implications of chronic exposure to sweetened aerosols in the respiratory system.

## 2. Materials and Methods

Articles for this review were selected using a structured and transparent methodology. A comprehensive literature search was conducted across major scientific databases, including Web of Science, Scopus, and PubMed, using targeted keywords related to the use of sweeteners in e-liquids, their oral intake and inhalation metabolisms, environmental fate, ecotoxicity, genotoxicity, oxidative stress, and recycling.

Inclusion criteria focused on the relevance to the review topic, methodological rigor, and publication in peer-reviewed journals. To ensure a thorough and up-to-date overview of the field, additional references were identified through citation tracking. Following the initial screening of titles and abstracts, full-text articles were evaluated to confirm their relevance and quality. Non-peer-reviewed manuscripts, including preprints and short conference papers, were excluded until the peer-review, necessary to assure research quality, is finished.

This systematic approach ensured the inclusion of high-quality studies published during the last 20 years, providing valuable insights into the current advances and ongoing challenges in the use, flavoring, and biological activity of sweeteners in e-cigarettes.

## 3. E-Cigarette Sweetening Overview

Both natural and synthetic sweeteners may be used to sweeten e-cigarette liquid and aerosol. Nevertheless, little to no investigation of their influence in electronic cigarette general safety, general toxicity, and ecological impact has been carried out.

### 3.1. Natural Sweeteners

Natural sweeteners added to e-cigarettes are generally those based on monosaccharides and include erythritol, neohesperidine, xylitol, and sorbitol, among other substances [51,52,53,54,55,56,57,58,59,60]. Although they are generally considered safer than the synthetic sweeteners, described in Section 3.2, their presence in electronic cigarettes, where they are inhaled nasally or orally in the presence of non-nutrient substances and not taken by mouth, alters their metabolic path (inclusively by the indigenous microbiome), which is why the investigation of their metabolic and environmental fate becomes of high importance.

The work [51] describes the impact of e-cigarette aerosol on the indigenous oral microbiome. Both natural and synthetic components possess cytotoxic effects on either epithelial cells or both indigenous and exogenous microbiota, leading to periodontal diseases. Moreover, the aerosol metabolism by-products strongly depend on bacterial community composition and include ethyl-4-acethylthiobutyrate, which is a frequent erythritol metabolite. Also, a quorum-sensing regulated gene expression in oral biofilms has been confirmed.

In [52], e-cigarettes containing the natural sweetener xylitol and synthetic sweetener sucralose, alongside their metabolites in aerosol, were analyzed by GC–MS. The e-cigarettes of the Velo brand, commercialized in Pakistan, were found to possess significant concentrations of xylitol, up to 16 mg/pouch, which were higher than those of some standard flavoring compounds, making it the expressive feature of the Velo brand. Moreover, in some cases, the sweetener declared concentration did not correspond to the real one, which might lead to a misinterpretation of the security perception of the vaping liquids.

The work [53] mentions the natural sweetener perillartine, used mostly in Japan as a component of Puff Bar Grape e-cigarette aerosol, which is similar to ethyl maltol. Both of them were used as taste correctors and may augment the nicotine biological action in the organism. The authors alert US federal regulators to impose the restrictions regarding classical cigarettes to e-cigarettes in order to prevent the legislative lacuna.

The work [54] mentions the simultaneous use of maltol, ethyl maltol, and ethyl acetate in e-cigarettes, aimed to hide not only the real flavor of nicotine and related compounds but also their smell. In this case, the natural sweeteners are used alongside the natural odorants, but the aldehydes, formed during their partial thermal decompositions, may be highly toxic.

The work [55] mentions the use of mogroside V from *S. grosvenorii*, a sweetener first used in Chinese cuisine and traditional medicine, in e-cigarettes as a flavoring agent. Mogroside V has shown a relatively safe behavior, so technically, this could be a viable alternative to the synthetic sweeteners mentioned in Section 3.2 for use in e-cigarettes, as it is thermally stable up to 150 °C, whereas sucralose decomposes at 120 °C. Nevertheless, the difference in the metabolism of oral and inhaled mogroside has to be taken into account.

The patent [56] mentions the sweetener for e-cigarette liquids based on the natural polyphenolic sweetener neohesperidine dihydrochalcone and the synthetic sweetener neotame. Nevertheless, the use of the natural sweeteners alongside the synthetic ones may alter the metabolism of both in the human organism and also provoke the synergic toxic effect between the two. Moreover, the oxidative stress provoked by neotame will concur with the antioxidant effect of the polyphenolic natural sweetener, and their efficiency will depend on the concentrations of both. The data of the permitted concentration range of both of the sweeteners are still pending a thorough investigation.

Common sweeteners, including glucose, sugar, fructose, and sorbitol, are rarely used in electronic cigarettes due to their dehydratation to furfural derivatives, which have already been detected in e-cigarette aerosol [57,58], and the influence of nicotine and other *N. tabacum* and *N. rustica* alkaloids on sugar consumption and insulin secretion, leading to the appearance of diabetes type 1 and 2 and amino acid metabolism deviation [59,60]. For this reason, they cannot be used as a safe flavoring component in e-cigarettes. General data of the use of natural sweeteners in electronic cigarettes are shown in Table 1.

So, natural sweeteners are already used in e-cigarettes as an alternative or additive to the artificial sugar substitutes, although their use is still less common. Nevertheless, there is a difference in the metabolic paths of these substances

- When heated;
- When inhaled vs. taken orally;
- In the presence of other substances.

The main factors influencing the biological action of the sweetened e-liquids are the solution composition, temperature, puff duration, and thermal stability of the sweetener used (see Appendix A and Appendix B). The temperature in vape aerosol on its way to the user’s respiratory tract may be enhanced by up to 145–150 °C; most of the synthetic and natural sweeteners partly degrade at that temperature, along with some of the other e-liquid components.

Substituted furfural and other toxic or conditionally toxic aldehyde derivatives are formed during vaping following degradation. For this reason, the development of regulatory norms concerning the use of sweeteners in e-cigarettes is necessary to prevent possible intoxication and stress [47,48].

In this case, the most efficient alternatives to artificial sweeteners are perillartine and mogroside V. As vaping temperature may rise up to 150 °C, at which most of the natural and certain synthetic sweeteners are partially degraded, these compounds have thermal stability advantages. Mogroside remains stable up to 150 °C, and perillartine melts at 102 °C without decomposition and may withstand higher temperatures up to 180°. For this reason, these compounds may be efficiently used in electronic cigarettes, being an efficient substitution for natural and synthetic sweeteners.

The compressed safety data of the sweeteners used in e-liquids are given in Appendix B.

### 3.2. Artificial Sweeteners

Artificial sweeteners are more common in electronic cigarettes than natural ones due to the presence of solvents in which the synthetic sugar substitutes are more soluble. Moreover, these substances generally have an intensively sweet taste, which hides the nicotine and *N. tabacum* alkaloids flavor and smell; however, their adsorption is enhanced, and their metabolism is modified. The artificial sweeteners most used in electronic cigarettes are aspartame, saccharin, acesulfame K, neotame (see [56] and Appendix B), and especially sucralose [52,61,62,63,64,65,66,67,68,69,70,71,72,73,74]. Among the sweeteners, sucralose is the most used in e-cigarettes, which is the reason why its addition to vaping products is revised in a separate subsection.

#### 3.2.1. Sucralose in E-Cigarettes

**Figure 3:**
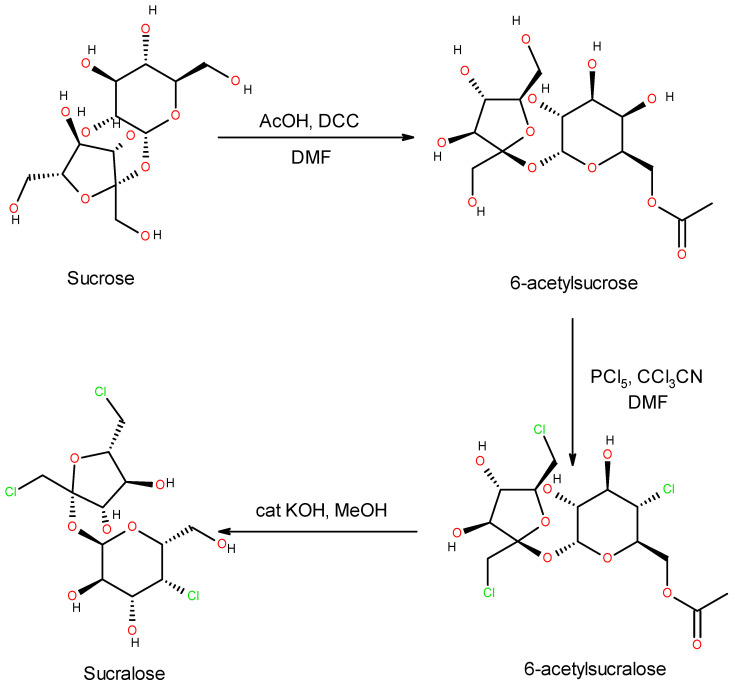
Sucralose synthesis from sucrose.

The reason why sucralose is the most frequently used in electronic cigarettes is its expressively sweet taste without a bitter aftertaste. This perception difference between sucralose and other synthetic sweeteners is explained by its carbohydrate nature, as it is in fact a trichlorogalactosucrose, industrially obtained from sucrose [35,62] (Figure 3).

Nevertheless, despite the expressively sweet taste of sucralose, its biological action in e-liquids, and also the toxin release promoted by it, makes its inhalation in vapes not so sweet. The oxidative stress following ROS formation, adduct formation, clastogenesis, direct and indirect DNA alteration, possible glycation, and environmental impact of sucralose have been reviewed in [35] and references therein. The present study complements the review with an emphasis on e-cigarettes.

Nevertheless, the main disadvantage of sucralose is its thermal instability in vaping conditions. At temperatures over 119 °C, it releases water, hydrogen chloride, and chloroorganic compounds, which are even more toxic than sucralose, broadening the gamma of negative activities of sucralose-based e-liquids.

A study by Schlapack, focused on e-liquids [63], examined the thermal degradation of sucralose when used as a sweetener in vaping products. The authors highlighted that several sucralose dehydration products are either carcinogenic or genotoxic. The inhalation route of exposure may present different biological impacts compared to ingestion, raising concerns about the potential synergistic genotoxic effect between sucralose and its degradation products. This negative biological activity is not the unique sucralose impact in electronic liquids.

Hu et al. [64] found that sucralose presence enhances the toxicity of benzo(a)pyrene, a component of either traditional cigarette smoke or e-cigarettes, in mice by inhibiting PGP-mediated efflux, leading to compound accumulation, increased ROS, and greater direct and indirect mutagenesis. Additionally, alterations in renal tissue structure were observed, leading to grave kidney failures.

Duell et al. [65] investigated the impact of sucralose and its degradation products on the stability and safety of glycerol- and propyleneglycol-based e-liquids by ^1^H NMR spectroscopy, IC, and GC–MS. The presence of sucralose and its degradation products in heated vaping liquids led to enhanced dehydratation and oxidation of solvents, leading to the enhancement of the concentrations of aldehydes and hemiacetal, confirmed by NMR spectroscopy. The harmful chloroorganic compounds, especially chlorohydrins, have also been detected. This leads to the conclusion that the sucralose in e-liquids, while it degrades, enhances the solvents’ thermal dehydratation rate. This has also been observed by Kerber [66].

Rosbrock et al. [67] analyzed the relation between the nature of vaping devices used for e-cigarette smoking, sucralose concentration, and taste perception of the aerosol. Four commercial vaping liquids without and with sucralose (1% *w*/*v*) using a cartridge and tank were analyzed. Sweetness and flavor intensity were more strongly felt when the cartridge was used, and olfaction was permitted, which leads to the conclusion that, in the case of a cartridge used as an aerosol outlet, the sucralose concentration becomes higher in the aerosol.

Recent 2024-dated research by Yan [68] with coauthors investigates the impact of sucralose and neotame on the metal precipitation from e-liquids, resulting in direct and indirect cyto- and genotoxic action. The propyleneglycol-based electronic liquids with 2% (*w*/*w*) and 5% neotame or sucralose were investigated by a CCK-8 assay. It was shown that 5% sucralose provoked the most intensive transition metal precipitation from the e-liquid, especially nickel, whereas neotame did not show such an impact. This effect was explained by chloride-ion formation during sucralose degradation (by HCl elimination and its interaction with metal), followed by metal part corrosion, metal ion release, and metal deposition on the container surface. Cytotoxicity tests showed reduced viability of SH-SY-56 cells at nearly 65% and Beas-2B cells at nearly 63%. As a conclusion, neotame is suggested as a safer alternative to sucralose. Nevertheless, the biological effect of neotame in e-liquids is not so safe, as described below (see Section 3.2.2. and Appendix A and Appendix B).

**Figure 4:**
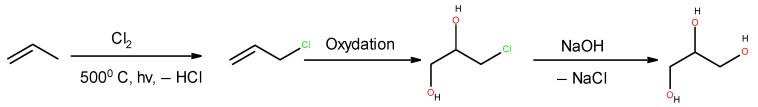
Glycerol classical synthesis from propylene. 3-chloropropane-1,2-diol is the oxidation product and glycerol precursor.

In [69], Moser et al. quantified by GC–MS/GC–FID and estimated the cytotoxic effects of sucralose degradation products. The most abundant of them is 3-chloropropane-1,2 diol, an extremely toxic compound, which is a precursor for glycerol synthesis from propylene (Figure 4).

3-chloropropane-1,2-diol was detected at concentrations up to 10 g/kg. Moreover, the metabolic activity of HUVEC/Tert2 cells was decreased significantly by e-liquid aerosol condensate. Therefore, the authors reinforce the recommendation against the use of sucralose in e-liquids. Nevertheless, the sucralose degradation route to 3-chloropropane-1,2 diol and then to glycerol might be efficiently used to eliminate sucralose from the environment via a circular economy approach.

El-Hellani et al. [70] mention sucralose as a component of menthol-flavored and nicotine-flavored electronic liquids and investigate the effect of sucralose on ROS generation, provoked by the components of vaping products. Sucralose increased ROS production, but this effect was manifested more intensively in the presence of nicotine base and not nicotinium cation.

Kim et al. [71] investigated the cariogenic potential of tooth surfaces exposed to the sweeteners in electronic cigarettes. It was seen that the contact of *S. mutans* with tooth enamel grew four times in the presence of sweetened e-liquid aerosol with sweeteners including sucralose, triacetin, ethylbutyrate, and hexylacetate. The percentage of enamel hardness loss for the control for them was also significant. Analogous results are also mentioned by Gaur in [72].

The work [73] by Maloney et al. investigates the comparison of the human abuse of unflavored and sucralose-sweetened electronic cigarettes. It has been shown that the addition of sucralose augmented e-cigarette human abuse potential (HAP) due to its more attractive taste. Moreover, the augmented adsorption of nicotine alkaloids in the presence of sucralose, cited earlier, may also contribute to this HAP increment.

In continuation of [66], the work [74] investigates the influence of common e-liquid flavorants and externally added nicotine to the formation of toxins in e-liquids. The statement of the enhanced toxic carbonyl compound formation in the presence of sucralose has been reinforced. Moreover, it has been detected that the presence of sucralose augments the aerosol aldehyde and ketone concentration due to either sucralose or filler decomposition (see also Appendix A and Appendix B).

The biological impact of sucralose added to e-liquids and their aerosols is shown in Table 2.

Therefore, despite the frequent use, sucralose in e-liquids and their aerosols manifest an ample repertoire of negative biological activity, including the degradation to toxic compounds, oxidative and metabolic stress, cariogenesis, and even an impact to human behavior, especially in devices in which the temperature is maintained higher than 119°.

For this reason, its use in e-liquids has to be limited or even banned [48,49,68,69], as the NOAEL and LOAEL of the degradation products are much lower than those of sucralose. Moreover, the exposure time factor, which was less important in the case of common cigarettes, also plays its role in the case of e-cigarettes.

The impact of the presence of other sweeteners on sucralose biological activity in e/liquids is still poorly understood. Nevertheless, there is evidence of the synergism of the toxical action of sucralose and other substances (sweeteners or not) [20,64].

Other synthetic sweeteners, despite being somehow safer, apparently have other types of peril, shown in the next subsection.

#### 3.2.2. Other Sweeteners in E-Cigarettes 

Contrarily to sucralose, other artificial sweeteners are less used in e-cigarettes, either due to their thermal instability or due to their bitter aftertaste [75,76,77,78,79,80,81,82,83,84], which becomes enhanced in the presence of nicotine products. Nevertheless, aspartame, neotame, saccharin, and acesulfame K are still used in e-liquids, and their use may even seem safer than that of sucralose [68], but their use implies certain objections.

The work [75], dated 2025, such as the works [76,77,78], mention the use of acesulfame K, aspartame, and saccharin, alongside sucralose, in e-cigarettes and smokeless tobacco products. The use of these three sweeteners in vaping products is rare (and the use of saccharin is nearly unique [77]) due to the possibility of methanol formation during the thermal and metabolic degradation of the mentioned substances. Moreover, there are some doubts concerning the safety of acesulfame K, aspartame, and saccharin to the genome and their possibility to cause intensive oxidative stress. Aspartame was also linked to certain cardiovascular events. The sweeteners most frequently used in e-cigarettes may be consulted in Appendix B.

Neotame [52,79] has been shown to be a hypothetically safe alternative to sucralose, aspartame, acesulfame K, and saccharin for use in e-cigarettes. Nevertheless, there are some objections about the influence of neotame to gut microbiota [80]. Moreover, such as in the case of aspartame, neotame is metabolized primarily by esterases, which yield methanol as a subproduct. Despite the small concentration of methanol produced during the metabolic process, the presence of other toxic components of the vape may generate a cumulative and synergetic effect. Another factor to be considered is the time-dependent realization of oxidative stress caused by these sweeteners, which becomes more intense in the presence of tobacco alkaloids [82,83,84]. For this reason, the FDA has only approved neotame in food, not for inhalation.

## 4. Discussion

The use of both natural and synthetic sweeteners in electronic cigarettes, despite the clear intention to alleviate or hide the *N. tabacum* or *N. rustica* alkaloids taste and smell for the addicted user, bears certain problems not only for the proper smokers but also for the environment. The genomic safety also becomes posed in peril.

For example, the use of sucralose in electronic cigarettes is still the most frequent, despite the ample variety of dangerous biological activity (up to the enhancement of the psychological addiction) it may pose. It is explained by purely perceptive and organoleptic properties. Nevertheless, the oxidative stress, genomic and environmental impact, and enhancement of cytotoxicity of this substance are really important. Moreover, sucralose is not thermally stable at vaping conditions, which may reach 150 and even more degrees centigrade, being decomposed into products that are even more poisonous. For this reason, the use of sucralose in e-cigarettes and vapes should be limited if not banned [68,69].

Aspartame, neotame, saccharin, cyclamate, and acesulfame K could serve as possible synthetic alternatives to sucralose. Nevertheless, their metabolism in the presence of nicotine alkaloids and when inhaled is still poorly investigated. The aspartic acid derivatives neotame and aspartame metabolize yielding methanol, which, even in small concentrations, may enhance the toxic action of other vape components. Moreover, some of them may be associated with carcinogenesis and cardiovascular diseases. For this reason, the use of these sweeteners must be considered with caution.

Even natural sweeteners, including carbohydrates, phenolic compounds, and amino acid derivatives, may be dangerous in the e-cigarette condition due to their thermal instability. The special cases among them are perillartine (an unsaturated aldoxime, nearly exclusive to Japan) and mogroside V (trisaccharide terpenic derivative), which are thermally more stable than the rest of the sweeteners and more sweet than some of them. For this reason, the use of perillartine and mogroside V, which are of natural origin and most thermally stable in the vaping conditions, as an alternative to other sweeteners in the dangerous vaping conditions, becomes viable.

## 5. Future Perspectives of the E-Cigarette Sweetening Investigation

The safety aspect of the use of sweeteners in electronic cigarettes is still under a thorough investigation, due to the high uncertainty about the following:

- The comparison of the biological action of taken and inhaled sweeteners due to the metabolic differences;
- The metabolic profile of the sweeteners in the presence of each other and their influence on the biological activity of standard cigarette and cigarette smoke components, including *N. tabaccum* alkaloids, and their decomposition and combustion products, including polycyclic arenes and hetarenes [85,86]. This interaction may be synergetic, summary, or antagonic, and it is certainly concentration dependent.

Therefore, the sweetener determination in electronic cigarette liquids and their aerosol is really up-to-date [87,88,89,90,91,92,93,94]. Spectrophotometric, chromatographic, and, sometimes, electrochemical methods may be applied to it.

As for 2025, the biological action of the sweetener or a couple of them in the presence of standard tobacco smoke toxins is still poorly studied, although some of the studies are still worth mentioning.

Tian et al. [95] exposed Sprague–Dawley rats to a xylitol-based aerosol for 90 days. The xylitol caused temporary hematological and bronchoalveolar alterations, which were reverted after the 28-day recovery period. The NOAEL of inhaled xylitol for rats was calculated as 2,9 mg/L, indicating the relative safety of inhaled xytitol as it is but not in the presence of e-cigarette components, which still needs to be discussed.

Kassem et al. [96] investigated the chemical safety of waterpipe tobacco smoking, which is somehow similar to e-cigarettes, as the WPT is also aromatized and sweetened. Both natural and synthetic sweeteners are found in WPT, being responsible directly or indirectly (as their thermal dehydratation products) on the augmentation of carcinogenesis, DNA alterations, and oxidative stress. For this reason, the presence of the sweeteners in WPT also has to be regulated by state organisms.

Afolabi [97] compared traditional tobacco smoking (TTS} with the use of e-cigarettes. Both of them have their specific toxic effects, and whereas TTS products are well studied, the health effect of e-liquids is still a concern. The flavorants and sweeteners added to e-cigarettes may be responsible for neuroinflammation, oxidative stress, CNS impacts, and reprotoxicity. Therefore, the influence of e-liquids with and without sweeteners has to be thoroughly studied in order to estimate their spermo- and ootoxic effects.

The review article [98] mentions that ethyleneglycol, propyleneglycol, and vegetal glycerol, and also their dehydratation products, do influence negatively the homeostasis of the epithelial cells. Moreover, the sweeteners may dehydratate, yielding the same and similar compounds, which might enhance this influence. For this reason, the sweeteners’ behavior in e-cigarettes during vaping might be extensively studied.

In summary, while sweeteners are generally recognized as safe for oral consumption (with certain limitations), their behavior and biological impact upon inhalation remain far from fully understood. The current body of evidence suggests that thermal decomposition, aerosolization, and interactions with other e-liquid constituents may significantly alter their toxicological profiles. These processes can lead to the formation of reactive or even carcinogenic intermediates, which may potentiate oxidative stress and inflammation in respiratory tissues. Moreover, the potential synergistic effects between sweeteners and nicotine, propylene glycol, glycerol, or flavoring agents necessitate further multidisciplinary research integrating toxicology, analytical chemistry, and molecular biology. Until comprehensive inhalation studies and standardized regulatory assessments are conducted, the use of sweeteners in e-cigarettes should be approached with caution, as their long-term health consequences cannot yet be reliably predicted.

## 6. Conclusions

From this brief review of the use of natural and artificial sweeteners as correctors of organoleptic and sensorial properties of e-liquids and their aerosols, it is possible to conclude that both natural and synthetic sweeteners (sugars and sugar substitutes) are added to vape liquids for the pure purpose of smell and taste correction, taking in mind neither the biological action of these substances and their metabolites nor their influence on the stability of cellular structure and even vaping equipment.

Sucralose is the most used sweetener in e-cigarettes. Nevertheless, its use in the vaping condition is highly compromised by both thermal instability and its chloroorganic nature. As a result, various negative influences to the genome (micronuclei formation, clastogenesis, DNA glycation, ROS-provoked DNA changes), environment, human metabolism, and even human behavior force the use of sucralose in electronic cigarettes to be restricted.

Synthetic sweeteners (aspartame, saccharin, neotame, acesulfame K, and cyclamate) are more rarely used in e-liquids. Although they may seem less dangerous than sucralose, their metabolism may yield toxic and conditionally toxic compounds and give a negative effect to well-being.

Even natural sweeteners are mostly unstable in the vaping condition, being thermally degraded by dehydratation. This statement includes sugars, polyphenolic compounds, ethers, and esters, which dehydratate yielding toxic carbonyls. For this reason, the most viable sweeteners for e-cigarettes as of 2025 seem to be perillartine and mogroside V, as they are natural and thermally stable. Nevertheless, the lacuna formed by the lack of state regulation on the use of the sweeteners in electronic cigarettes must be fulfilled.

## Figures and Tables

**Table 1:** Natural sweeteners in e-liquids and their biological actions.

Natural Sweetener	Nature of Substance	Objections	Nature of the Study	CAS Number:	Reference
Erythritol, xylitol	Carbohydrate-derived polyol	Cytotoxicity, Partial dehydratation, Metabolism, dependent on gut microbiota	In vivo, in vitro	149-32-6, 87-99-0	[51]
Xylitol (in the presence of synthetic sucralose)	Carbohydrate-derived polyol (involving carbohydrate-derived chlorohydrin)	Sweetener concentration, non-correspondent to the declared, possible metabolic synergetic effect between natural and artificial sweetener, sucralose, and xylitol dehydratation and its products	In vitro	87-99-0, 56038-13-2	[52]
Perillartine, ethylmaltol	Oxyme of an unsaturated aldehyde, in the presence of an ether	Sweetener, used mostly in Japan. Possible substitution of sucralose and other artificial sweeteners. Hydroxylamine formed during metabolism in some people. Dehydratation of ethylmaltol	In vitro	30950-27-7, 4940-11-8	[53]
Maltol, ethylmaltol, ethylacetate	Carbohydrate-derived polyol and its ether in the presence of a common ester	Decomposition, yielding toxic aldehydes	In vivo	4940-11-8, 118-71-8, 141-78-6	[54]
Mogroside V	A trisaccharide derivative of a terpenoid compound	Thermal stability. Possible substitute of sucralose. Objections about metabolism of inhaled form	In vitro	88901-36-4	[55]
Neohesperidine dihydrochalcone (in the presence of neotame)	Polyphenolic compound (an aromatic amino acid derivative being involved)	Synergetic toxic effect of inhaled form. Concurrence between the antioxidant action of neohesperidine and the oxidative stress of neotame	In vitro	13241-33-3, 165450-17-9	[56]
Glucose	Carbohydrate	Glucose intake and insulin secretion, affected by *Nicotiana* alkaloids. Dehydratation products involved	In vivo	50-99-7	[57]
Sugar	Carbohydrate	Glucose intake and insulin secretion, affected by *Nicotiana* alkaloids. Dehydratation products involved	In vivo	57-50-1	[58]
Fructose	Carbohydrate	Dehydratation products include furfural derivatives and aldehydes, which may be toxic when inhaled	In vivo	57-48-7	[59]
Sorbitol	Carbohydrate-derived polyol	Dehydratation leading to toxic compounds in aerosol	In vivo	50-70-4	[60]

**Table 2:** Marketing and biological aspects of use of sucralose in e-liquids (↑—increase, ↓—decrease).

Vaping Products	Biological Activity	Experiment Type	Reference
E-liquid (in the presence of neotame)	Sweetener concentration, non-correspondent to the declared, possible metabolic synergetic effect between natural and artificial sweetener, sucralose and xylitol dehydratation and its products, ↑ toxic carbonyl compounds	In vitro	[52]
E-liquid aerosol	↑ toxic chloroorganic degradation products	In vitro, in vivo	[63]
Benzo[a]pyrene exposure	↑ renal toxicity, PGP inhibition, ↑ ROS	In vivo	[64]
E-liquid and e-liquid aerosol	↑ sucralose degradation rate, ↑ solvents oxidation and dehydratation rate, ↑ toxic chloroorganic degradation products, ↑ toxic carbonyl compounds	In vitro	[65]
E-liquid	↑ sucralose degradation rate, ↑ solvents oxidation and dehydratation rate, ↑ toxic chloroorganic degradation products, ↑ toxic carbonyl compounds	In vivo	[66]
E-liquid and e-liquid aerosol	↑ flavor perception in cartridge aerosol	In vivo	[67]
E-liquid and e-liquid aerosol	↑ heavy metal precipitation, ↑ metallic parts corrosion, ↑ cytotoxic effect ↓ cells viability, neotame suggested as a safer alternative to sucralose	In vitro	[68]
E-liquid and e-liquid aerosol	↑ toxic chloroorganic degradation products, ↑ toxic carbonyl compounds, ↓ cells’ metabolic activity, sucralose ban in e-liquids suggested	In vitro	[69]
E-liquid and e-liquid aerosol	↑ ROS by different manner, depending on nicotine formulation	In vivo	[70]
E-liquid aerosol	↑ enamel contact, ↑ cariogenesis, ↓tooth hardness	In vivo	[71]
E-liquid	↑ enamel contact, ↑ cariogenesis, ↓tooth hardness	In vivo	[72]
E-liquid and e-liquid aerosol	↑ HAP	In vivo	[73]
E-liquid	↑ sucralose degradation rate, ↑ solvents oxidation and dehydratation rate, ↑ toxic chloroorganic degradation products, ↑ toxic carbonyl compounds	In vitro	[74]

**Figure A1:**
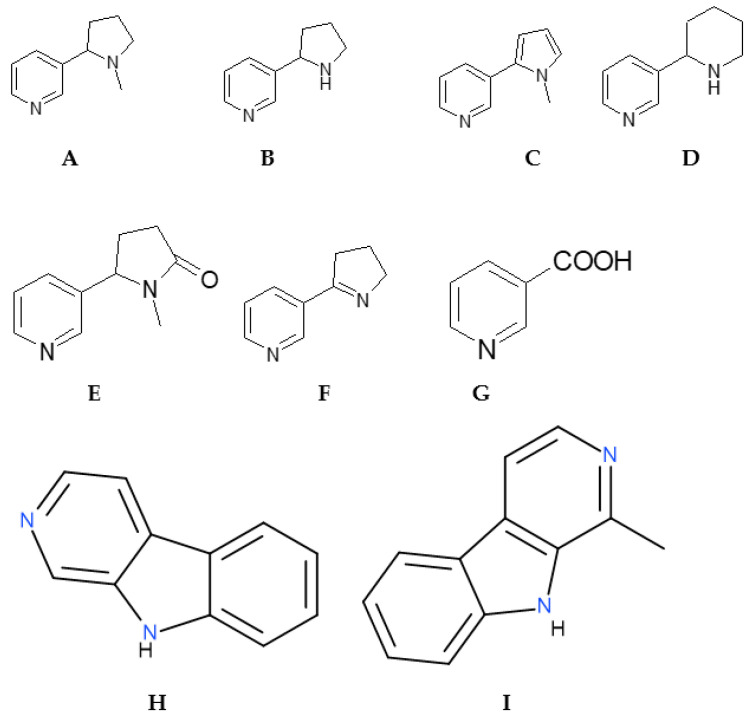
Nicotine (**A**) and its analogs nornicotine (**B**), nicotirine (**C**), anabasine (**D**), cotinine (**E**), myosmin (**F**), nicotinic acid (**G**), norharmane (**H**), and harmane (**I**).

**Figure A2:**
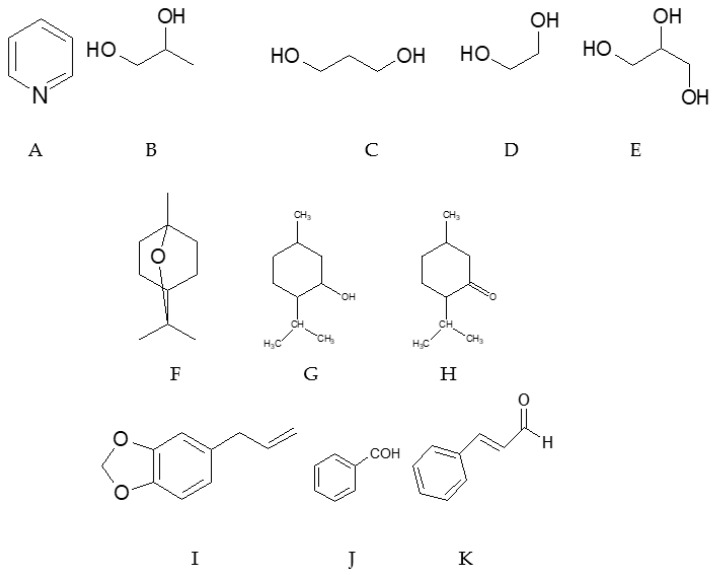
The main e-cigarette components pyridin (**A**), propyleneglycol (**B**), trymethyleneglycol (**C**), ethyleneglycol (**D**), glycerol (**E**), eucalyptol (**F**), mentol (**G**), menthone (**H**), safrol (**I**), benzaldehyde (**J**), and cynnamic aldehyde (**K**).

**Figure A3:**
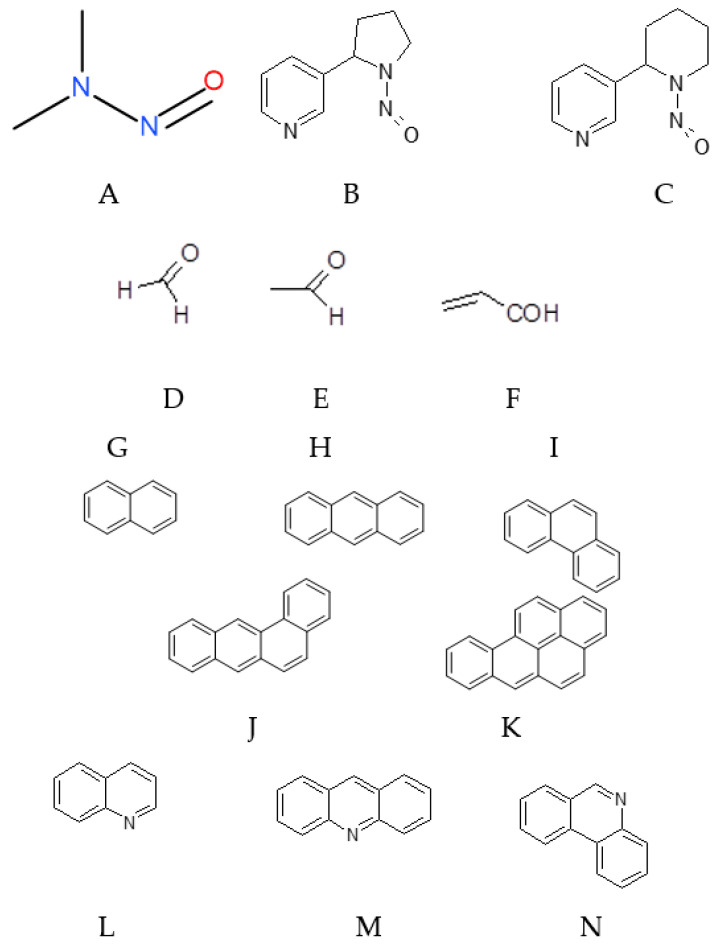
The most important adverse components of e-liquids: N-nitrosodimethylamine (**A**), N-nitrosonornicotine (**B**), N-nitrosoanabasine (**C**), formaldehyde (**D**), acetaldehyde (**E**), acroleine (**F**), naphthalene (**G**), anthracene (**H**), phenanthrene (**I**), pyrene (**J**), benzo[a]pyrene (**K**), quinoline (**L**), acridine (**M**), phenanthridine (**N**).

## Data Availability

No new data were created or analyzed in this study. Data sharing is not applicable to this article.

## Abbreviations

The following abbreviations are used in this manuscript:

IC	Ionic Chromatography
GC–MS	Gas Chromatography coupled with Mass Spectroscopy
ROS	Reactive Oxygen Species
HAP	Human Abuse Potential
C CNS	Central Nervous System
WPT	Waterpipe Tobacco Smoking
TTS	Traditional Tobacco Smoking

## Appendix A. E-Cigarette Chemical Composition

### Appendix A.1. Brief Overview of Electronic Cigarettes

Electronic cigarettes (commonly referred to as e-cigarettes or vapes) are devices designed to deliver nicotine and other chemical constituents through the inhalation of vapor rather than combustion products [99]. The device operates by heating a liquid, commonly known as e-liquid or vape juice, which contains nicotine alongside a complex mixture of other compounds. Upon heating, the liquid is vaporized, allowing its constituents to be inhaled into the nasopharynx. From both chemical–toxicological and physical perspectives, the mechanism of vapor generation in e-cigarettes shares notable similarities with traditional tobacco consumption, such as pipe smoking or chewing tobacco.

Structurally, an e-cigarette comprises a housing and a mouthpiece, within which an atomizer is embedded [100]. The atomizer typically incorporates a heating element, a lithium-ion battery, and a cartridge reservoir containing the e-liquid. Activation of the device initiates the evaporation of the liquid’s components, particularly volatile compounds, which are then delivered to the oral cavity and respiratory tract via the mouthpiece.

E-liquids may be formulated with natural or synthetic ingredients; however, even so-called “natural” products frequently include synthetic additives. These additives often serve to mask the characteristic odor of tobacco, a feature that has been identified as a key factor in the popularity of e-cigarettes among adolescents and young adults, enabling the concealment of nicotine use [101].

The chemical composition of e-cigarette liquids and their aerosols may vary and exhibits some similarities to conventional cigarette smoke but is distinguished by the presence of synthetic compounds and, in many cases, the replacement of natural constituents with artificial analogues. This substitution may exacerbate potential health risks, particularly in developing adolescents. Several e-liquid components have been characterized as having irritating, mutagenic, teratogenic, and carcinogenic properties [102].

This appendix aims to provide a comprehensive overview of the chemical constituents of e-cigarette liquids and their associated toxicological effects. Particular emphasis is placed on synthetic additives and their biological impact. Finally, this section offers an evidence-based assessment of the potential hazards associated with e-cigarettes, their liquid formulations, and inhaled aerosols, highlighting the risks posed to users, especially younger populations.

### Appendix A.2. Main Substances of the E-Liquids

#### Appendix A.2.1. Nicotine and Its Analogues

Tobacco smoke and e-cigarette liquids contain a variety of biologically active alkaloids [103,104,105,106], among which nicotine, nornicotine, nicotinic acid, anabasine, cotinine, and myosmin are the most significant (Figure A1). These compounds are primarily responsible for the addictive and toxic effects of tobacco products.

Nicotine, a tertiary amine and pyridine derivative, is the primary addictive component. Named after Jean Nicot, who introduced tobacco to France in the 16th century, it was first synthesized in 1904. Nicotine exerts its effects by interacting with nicotinic acetylcholine receptors at nerve endings, causing cation influx (Na^+^, K^+^, Ca^2+^), triggering nerve impulses, and activating the parasympathetic nervous system. This manifests as increased heart rate, enhanced secretion of saliva and sweat, intestinal peristalsis, and short-term stimulation of cognitive activity. Chronic exposure leads to psychological and physical dependence as well as metabolic and cellular changes contributing to cardiovascular disease, cancer, teratogenic effects, and mutagenesis.

Nornicotine, the demethylated precursor of nicotine, has a secondary amine group that increases its receptor activity, making it potentially more potent than nicotine in stimulating the brain.

Nicotinic acid (niacin, vitamin B_3_) is a less basic derivative and a metabolic product of nicotine and related alkaloids. While essential for human metabolism, its formation from tobacco alkaloids involves toxic intermediates, making direct intake via smoking inadvisable.

Anabasine is an isomer of nicotine with strongly basic properties and similar biological activity. It can form ion salts (anabasinium) that mimic nicotine salts and, in combination with nicotine, may exacerbate toxicity.

Cotinine, a primary metabolite of nicotine, has milder effects on the body and is used as a biomarker of tobacco exposure. Its retention is influenced by additives such as menthol.

Myosmin has intermediate activity between nicotine and nicotinic acid, stimulating dopamine and adrenaline release and activating the parasympathetic nervous system. Its metabolism produces highly toxic intermediates, increasing the potential hazards of e-cigarette liquids containing this alkaloid.

The main product of the oxidation of these substances is nicotinic acid, a substance important for human life. It is known to us as vitamin B 3. However, the process of forming nicotinic acid from the aforementioned substances is a multi-stage process, the intermediates of which are extremely toxic.

Norharman and harman are both neuroactive β-carboline alkaloids from *N. rustica*, a class of naturally occurring compounds found in plants and, notably, in many thermally processed foods like coffee, roasted meat, and tobacco smoke. Harman has a methyl group at the 1-position, making it 1-methyl-9H-pyrido [4-b]indole, while norharman is the simpler 9H-pyrido [4-b]indole structure. They have a wide range of biological effects, including acting as inhibitors for the enzyme monoamine oxidase (MAO).

Overall, the chemical profile of e-cigarettes mirrors that of traditional tobacco products, but differences in concentrations, especially with nicotine salts and other alkaloid-rich formulations, can increase toxicological risks. A joint table for the nicotine and analogous compounds found in e-cigarettes is shown in Table A1.

**Table A1:** Nicotine analogues commonly found in e-cigarettes.

Compound	CAS Number	Typical Concentration	Mechanism of Action/Biological Effects	Approx. LD_50_ (Oral, Rat)	Notes
**Nicotine**	54-11-5	Cigarettes: ~1 mg/cig; E-liquids: 0–60 mg/mL	Tertiary amine; binds nicotinic acetylcholine receptors; stimulates parasympathetic NS; increases HR, secretion, peristalsis; highly addictive; carcinogenic, mutagenic, teratogenic	50 mg/kg	Nicotine salts increase receptor saturation and systemic absorption; main addictive component
**Nornicotine**	65-99-0	Cigarettes: 0.1–0.2 mg/cig; E-liquids: trace–5 mg/mL	Secondary amine; stronger receptor activity than nicotine; excitatory; additive toxicity	Not well-established	Demethylated nicotine; more potent CNS effects
**Nicotinic acid (Niacin, Vitamin B_3_)**	59-67-6	Cigarettes: trace; E-liquids: trace	Metabolic cofactor for NAD/NADP; minimal receptor activity; formed from oxidation of nicotine/nornicotine	900 mg/kg	Toxic intermediates formed during metabolism from tobacco alkaloids
**Anabasine**	494-52-0	Cigarettes: 0.2–0.5 mg/cig; E-liquids: trace	Nicotine isomer; forms anabasinium salts; mimics nicotine activity; addictive	11 mg/kg	Potentiates nicotine toxicity; present in trace amounts
**Cotinine**	486-12-4	Smokers’ blood: 250–300 ng/mL; E-liquids: <1–10 mg/mL	Nicotine metabolite; weaker CNS stimulation; biomarker of tobacco exposure; mild antidepressant effect	>400 mg/kg	Retention prolonged by menthol; marker of passive smoking
**Myosmin**	512-12-1	E-liquids: variable, up to 1–5 mg/mL	Stimulates dopamine and adrenaline release; activates parasympathetic NS; metabolizes to highly toxic intermediates	Not well-established	Increases toxicity when enriched in e-liquids
**Norharman (β-carboline)**	1656-85-1	*N. rustica*: 0.2–0.5 mg/g dry leaves; trace in e-liquids	MAO inhibitor; neuroactive; may potentiate nicotine effects; mutagenic and carcinogenic potential	100 mg/kg	Found mainly in *N. rustica*; contributes to addiction and CNS effects
**Harman (β-carboline)**	123-32-0	*N. rustica*: 0.5–1 mg/g dry leaves; trace in e-liquids	MAO inhibitor; neuroactive; can potentiate nicotine; mutagenic, teratogenic, and carcinogenic	120 mg/kg	Structurally similar to norharman; synergizes with nicotine for addictive potential

#### Appendix A.2.2. E-Liquid Fillers

E-cigarette solutions are primarily in the liquid phase, but their individual components may exist as liquids or crystalline substances dissolved within the filler. To achieve solubility, polyols with or without pyridine are typically employed [107,108,109] (Figure A2).

Pyridine, an aromatic nitrogen-containing compound, is used in trace amounts to dissolve nicotine and its analogues, following the principle “the similar compound dissolves the similar compound”. Despite its small concentration, pyridine is highly toxic. Inhalation irritates the mucous membranes of the upper respiratory tract for several hours and can cause mild euphoria, suffocation, weakness, vomiting, or, at high exposures, loss of consciousness, hypotension, and bradycardia. Oral intake can interfere with vitamin B_1_ metabolism.

Unlike pyridine, the main solvents in e-cigarette liquids are polyhydric alcohols, such as 1,2-propanediol (propylene glycol), 1,3-propanediol (trimethylene glycol), ethylene glycol, and glycerin. Their toxic profiles differ substantially: propylene glycol and glycerin are generally safe in small doses, whereas ethylene glycol is highly toxic. Ethylene glycol is metabolized to aldehydes and acids, producing symptoms similar to alcohol intoxication initially, which can progress to nausea, vomiting, agitation, impaired consciousness, tetany, and even coma.

Polyols can react with carboxylic acids to form esters, which often have pleasant odors or flavors. These compounds, both natural and synthetic, are commonly added to e-liquids to mask the characteristic odor of nicotine. Examples include eucalyptol, used for rosemary flavoring, which exhibits antibacterial properties at low doses but can become highly toxic at elevated concentrations. Menthol and its oxidized form menthone, derived from mint, reduce allergic sensitivity and enhance the palatability of smoke. Menthol is also widely used in pharmaceuticals, cosmetics, and food products.

Safrole, a cyclic phenolic ether with a pleasant odor, is toxic, mutagenic, and carcinogenic. Aldehydes and ketones, such as benzaldehyde and cinnamic aldehyde, contribute fruity or cinnamon-like flavors but are highly toxic. Benzaldehyde is carcinogenic and mutagenic, whereas cinnamic aldehyde can cause lung epithelial inflammation and bronchiolitis obliterans.

In summary, e-cigarette liquids contain solvents and flavorings that can be conditionally safe at low concentrations but become hazardous at higher doses. Their primary function—masking the smell of nicotine—can, therefore, come at the cost of systemic and respiratory toxicity. The data about the most common filler components are shown in Table A2.

**Table A2:** E-liquids main filler components.

Compound	CAS Number	Typical Concentration in E-Liquids	Biological/Toxic Effects	Thermal Decomposition/Toxic Products	Notes
**Pyridine**	110-86-1	Trace (≤0.1%)	Irritates mucous membranes; inhalation: mild euphoria, suffocation, vomiting; high exposure: hypotension, bradycardia, loss of consciousness; interferes with vitamin B_1_ metabolism	On heating: forms nitrogen oxides, carbon monoxide, and pyridyl radicals; highly toxic	Used to dissolve nicotine and analogues; extreme caution required
**1,2-Propanediol (Propylene Glycol)**	57-55-6	30–70% of liquid	Generally safe as food additive (E1520); excessive inhalation: throat irritation, cough, minor CNS effects	Decomposes at high temperatures: acrolein, formaldehyde	Main solvent; contributes to vapor formation; relatively safe at standard e-cigarette temperatures
**1,3-Propanediol (Trimethylene Glycol)**	504-63-2	5–15%	Low toxicity; mild respiratory irritation possible	Thermal decomposition: acrolein, formaldehyde	Used as a thickener and vapor carrier
**Ethylene Glycol**	107-21-1	Trace–up to 5%	Highly toxic; metabolized to glycolaldehyde, glycolic acid, oxalic acid; causes nausea, vomiting, CNS depression, renal failure	Decomposes to formaldehyde, acetaldehyde, acrolein	Dangerous if present in high concentration; should be minimized
**Glycerin (Glycerol)**	56-81-5	10–60%	Generally safe; can cause cough or throat irritation	Thermal decomposition: acrolein, acetaldehyde	Major solvent; contributes to visible vapor; safe at moderate temperatures
**Eucalyptol (1,8-Cineole)**	470-82-6	Trace—1%	Mild antibacterial; excessive inhalation: CNS effects, nausea, hepatotoxicity	Decomposition: formaldehyde, CO, reactive terpenoids	Flavoring; overuse can increase toxicity
**Menthol**	89-78-1	0.1–2%	Cooling effect; reduces irritation; can induce mild CNS depression at high doses	Decomposition: menthone, reactive aldehydes, CO	Common in mint-flavored e-liquids; can be used as ethers/esters
**Menthone**	14073-97-3	Trace	Oxidized form of menthol; similar effects	Decomposition: reactive aldehydes, CO	Flavoring and aroma compound
**Safrole**	94-59-7	Trace	Mutagenic, carcinogenic; hepatotoxic	On heating: forms allylbenzene radicals, CO, formaldehyde	Natural aromatic ether; toxic even in small quantities
**Benzaldehyde**	100-52-7	Trace—0.5%	Fruity aroma; mutagenic, carcinogenic; irritates respiratory tract	On heating: benzoic acid, CO, benzene derivatives	Flavoring; risk increases with temperature
**Cinnamic Aldehyde**	104-55-2	Trace—0.5%	Cinnamon/clove aroma; irritates lung epithelium; can cause bronchiolitis obliterans	Decomposes to cinnamic acid, CO, aldehyde radicals	Strong flavoring; toxic at high concentrations or prolonged inhalation

It is important to mention some key notes on heating and aerosol formation.

Most solvents and flavoring agents can decompose when heated, producing highly reactive aldehydes (formaldehyde, acrolein), carbon monoxide, and free radicals.

The risk of acute and chronic toxicity increases with high-power vaping due to higher temperatures and prolonged exposure.

Even compounds considered “safe” in food (propylene glycol, glycerin) can form toxic intermediates when heated.

Masking the smell of nicotine is often achieved by flavoring compounds, which can become toxic when their concentrations are increased or during thermal decomposition.

#### Appendix A.2.3. Adverse e-Cigarette Components

In addition to primary tobacco alkaloids such as nicotine and its analogues, both conventional and electronic cigarettes contain adverse components [110,111,112] (Figure A3). These substances may form naturally in the tobacco plant, be added during manufacturing, or be entirely synthetic.

Nitrosamines are among the most potent carcinogens in tobacco smoke. The simplest, N-nitrosodimethylamine, can even be produced from partial oxidation of rocket fuel (1,1-dimethylhydrazine). In tobacco products, the most characteristic specific tobacco nitrosamines include N-nitrosonornicotine and N-nitrosoanabasine, formed from secondary amines in nicotine precursors.

Nitrosamines primarily affect the liver, potentially causing internal bleeding, hemorrhage, memory loss, and loss of consciousness up to coma. Even a single exposure can act as a mutagen, affecting the health of offspring. While trace nitrosamines may be present in fried foods, tobacco products are a far more significant source.

Toxic aldehydes, such as formaldehyde (methanal), acetaldehyde (ethanal), and acrolein (propenal), are present in e-cigarette liquids both from the tobacco itself and through oxidation of solvents or flavoring agents.

Formaldehyde is highly toxic, causing systemic weakness, respiratory failure, mucosal burns, nephritis, and, in severe cases, death. Acetaldehyde is moderately toxic, an irritant of the upper respiratory tract, and a Group I carcinogen; it is also produced during ethanol metabolism. Acrolein is extremely reactive due to its conjugated carbonyl and double bond; it irritates mucous membranes, induces mutations, and alters genetic material.

Other ecotoxic and carcinogenic compounds in fillers and smoke include polynuclear arenes, such as naphthalene, anthracene, phenanthrene, pyrene, and benzopyrene. The carcinogenicity increases with the number of fused aromatic rings, with benzopyrene being highly potent even at low concentrations due to DNA-reactive metabolites. Pyridine polycyclic derivatives, including quinoline, acridine, and phenanthridine, present similar or greater hazards.

Sulfur(IV) dioxide (SO_2_) is produced during combustion of sulfur-containing organic compounds (thiols, thiophenes) and is extremely toxic. Acute exposure causes runny nose, cough, hoarseness, sore throat, vomiting, and pulmonary edema.

Heavy metal ions, including Fe^2+^, Zn^2+^, Mn^2+^, Cd^2+^, Ni^2+^, and Cr^3+^, may be present in fillers from raw materials or manufacturing processes. These metals are highly toxic, and their concentrations in e-cigarettes can exceed those in conventional cigarettes, increasing the likelihood and severity of intoxication.

The data about the most common adverse components of e-liquids are shown in Table A3.

**Table A3:** E-liquids most common adverse compounds.

Compound	CAS Number	Typical Concentration	Biological/Toxic Effects	Thermal Decomposition/Toxic Products	Notes
**N-Nitrosodimethylamine (NDMA)**	62–75-9	Trace–μg/mL	Potent carcinogen; mutagenic; hepatotoxic; may cause internal bleeding, memory loss, coma	Decomposes to NOx, formaldehyde, and methyl radicals	Can form from nitrites and secondary amines; also a by-product of tobacco nitrosation
**N-Nitrosonornicotine (NNN)**	16,517-33-6	μg/cigarette; trace in e-liquids	Carcinogenic, mutagenic; liver toxicity; affects fetal health	Thermally stable but can form reactive nitrosyl species	Specific tobacco nitrosamine derived from nornicotine
**N-Nitrosoanabasine (NAB)**	16,517-34-7	μg/cigarette; trace in e-liquids	Carcinogenic; liver toxicity; mutagenic; neurotoxic	Can generate reactive nitrogen species when heated	Specific tobacco nitrosamine derived from anabasine
**Formaldehyde (Methanal)**	50-00-0	1–100 μg/puff	Highly toxic; irritant; carcinogenic; mucosal burns; nephrotoxic; respiratory failure	Further oxidizes to formic acid, CO, free radicals	Formed from oxidation of methanol, solvents, or glycerol/propylene glycol at high temperatures
**Acetaldehyde (Ethanal)**	75-07-0	1–50 μg/puff	Moderately toxic; irritant; carcinogenic (Group I); CNS effects; contributes to passive smoking irritation	Oxidizes to acetic acid; forms reactive aldehyde radicals	Produced from ethanol metabolism and tobacco combustion
**Acrolein (Propenal)**	107-02-8	0.5–20 μg/puff	Strong irritant; mutagenic; cytotoxic; used as tear gas; damages respiratory tract	Can polymerize or oxidize to acrylic acid, free radicals	Highly reactive α,β-unsaturated aldehyde formed during thermal decomposition of glycerol or polyols
**Polynuclear Aromatic Hydrocarbons (PAHs)**	Various (see below)	Trace–μg/puff	Carcinogenic; mutagenic; lipophilic; accumulates in tissues	Can oxidize to epoxides, quinones, ROS	Examples: Naphthalene (91-20-3), Anthracene (120-12-7), Phenanthrene (85-01-8), Pyrene (129-00-0), Benzopyrene (50-32-8)
**Quinoline**	91-22-5	Trace	Mutagenic, carcinogenic; neurotoxic	Forms reactive nitrogen species upon heating	Pyridine derivative with aromatic ring system; highly active electrophile
**Acridine**	107-18-6	Trace	Mutagenic; carcinogenic; DNA intercalator	Forms epoxides, radicals on heating	Pyridine derivative with planar aromatic structure
**Phenanthridine**	86-42-4	Trace	Mutagenic; carcinogenic; DNA intercalator	Forms reactive species	Pyridine derivative, highly reactive toward DNA
**Sulfur(IV) dioxide (SO_2_)**	7446-09-5	μg–mg/puff	Irritant of eyes, throat, respiratory tract; causes cough, hoarseness, pulmonary edema	Oxidation/reduction with other smoke components produces sulfites and radicals	Produced from combustion of sulfur-containing compounds (thiols, thiophenes)
**Fe^2+^, Zn^2+^, Mn^2+^, Cd^2+^, Ni^2+^, Cr^3+^**	Various	Trace–μg/mL	Heavy metal toxicity: nephrotoxic, hepatotoxic, carcinogenic, neurotoxic	Can catalyze oxidative reactions forming ROS	Present from raw materials and manufacturing; often higher in e-cigarettes than conventional cigarettes

## Appendix B. The General Safety Data for Sweeteners Used in E-Cigarettes

**Table A4:** Safety data for the most used sweeteners.

**Erythritol**	149-32-6	0.1–5%	Low-calorie sugar alcohol; mostly excreted unchanged; generally safe; may cause mild GI upset in excess	Can decompose at high temperatures to formaldehyde, acrolein	Polyol; contributes to sweetness and viscosity
**Sorbitol**	50-70-4	0.1–5%	Sugar alcohol; low-calorie; excessive intake can cause laxative effects	Thermal decomposition: formaldehyde, acrolein, organic acids	Often used to mimic sugar sweetness and as a humectant
**Xylitol**	87-99-0	0.1–5%	Sugar alcohol; anti-cariogenic; mild laxative in excess	Heating: formaldehyde, furfural, acetaldehyde	Common sugar substitute; safe in moderate doses in food but less safe if inhaled
**Mannitol**	69-65-8	0.1–5%	Sugar alcohol; diuretic at high doses; generally low toxicity	Thermal decomposition: formaldehyde, acrolein	Often used for sweetness and viscosity
**Neohesperidin dihydrochalcone (Neohesperetine)**	20702-77-6	Trace–0.1%	Non-caloric; sweetener; generally safe; may induce mild GI effects	Heating: phenolic decomposition products, aromatic aldehydes	Natural flavonoid derivative
**Perillartine**	3248-54-6	Trace–0.1%	Non-caloric sweetener; safe at low doses; bitter taste at high concentrations	Thermal decomposition: aromatic ketones, reactive aldehydes (at temperatures close to 200 °C)	Sweetener mostly used in Japan; thermally stable; very high sweetness potency
**Ethyl maltol**	4940-11-8	Trace–0.5%	Flavor enhancer; low toxicity; possible mild irritation	Thermal decomposition: furans, formaldehyde, acetaldehyde	Commonly used to enhance sweetness perception
**Mogroside V**	126530-29-9	Trace–0.5%	Natural sweetener from Siraitia grosvenorii; very low toxicity; non-caloric	Thermal decomposition at temperatures over 160^0^ C: glycoside hydrolysis products	High-intensity natural sweetener. Can be used in e-liquids due to relative thermal stability
**Glucose**	50-99-7	0.1–5%	Common sugar; high intake can raise blood sugar	Thermal decomposition: caramelization products, HMF (5-hydroxymethylfurfural), CO	Simple sugar; may caramelize at high temperature
**Sucrose (Sugar)**	57-50-1	0.1–5%	Common sugar; excessive intake affects glycemia	Thermal decomposition: caramelization, HMF, CO, aldehydes	Standard sugar; prone to thermal decomposition
**Fructose**	57-48-7	0.1–5%	Simple sugar; high intake can cause GI upset	Thermal decomposition: HMF, aldehydes, CO	High sweetness; forms toxic aldehydes at high temperatures
**Acesulfame K**	55589-62-3	Trace–0.5%	Non-caloric sweetener; generally safe; slight bitter aftertaste	Thermal decomposition: acetoacetic derivatives, formaldehyde	Stable under moderate heat; used in combination with other sweeteners
**Aspartame**	22839-47-0	Trace–0.5%	Low-calorie sweetener; metabolized to phenylalanine; unsafe for phenylketonuria	Decomposes at high temperature to diketopiperazine, methanol, phenylalanine	Heat-sensitive; commonly used in cold e-liquids. Yields toxic metabolites when inhaled. Metabolizes to methanol
**Saccharin**	81-07-2	Trace–0.5%	Non-caloric; safe at low doses; not metabolized	Can form aromatic decomposition products on heating	Stable; synthetic sweetener derived from toluene
**Sucralose**	56038-13-2	Trace–0.5%	Non-caloric; mostly excreted; heat-stable up to moderate temperatures	Decomposes to chlorinated aldehydes, dioxins, tetrachlorodibenzofurans	Resistant to metabolism; ecotoxic accumulation potential, oxidative stress, interaction with DNA, synergetic influence on the toxicity of other e-cigarette components
**Neotame**	165450-17-9	Trace–0.1%	Non-caloric sweetener; safe in small quantities; similar to aspartame but more stable	Can form diketopiperazine and related breakdown products on heating	Heat-stable compared to aspartame; very high sweetness potency but yields toxic metabolites when inhaled. Metabolizes to methanol
